# Supplementary material for: The Putative S1PR1 Modulator ACT-209905 Impairs Growth and Migration of Glioblastoma Cells In Vitro
Source: Cancers (Basel). 2023 Aug 26;15(17):4273. doi: 10.3390/cancers15174273 (PMC10486705; doi:10.3390/cancers15174273)
Supplement: Supplementary file 1 [file cancers-15-04273-s001.zip › Supplementery Figures S1-S5.pdf]

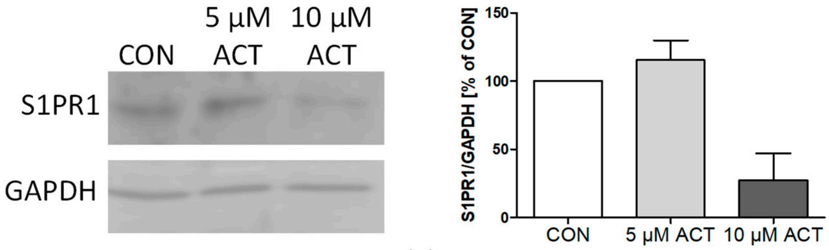

(a)

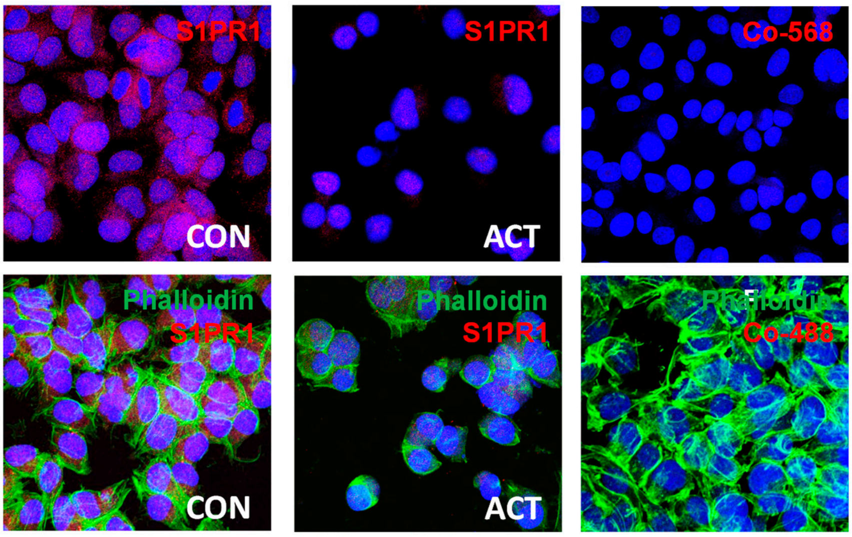

(b)

Supplemental Figure S1. S1PR1 protein expression after exposure to ACT209905. (a) Immunoblot analysis of LN-18 GBM cells treated for 72h with ACT-209905. The densitometric analysis is shown in the right panel with normalization to GAPDH as housekeeping protein (n=2). (b) Immunofluorescence analysis of S1PR1 protein in GBM cells. LN18 cells were incubated for 48h without (CON, A+D) or with 20 µM ACT (ACT, B+E). The cells were stained with a polyclonal anti-S1PR1 antibody (A, B, D, E) and secondary antibodies conjugated with *Alexa Fluor*®- 568 or *Alexa Fluor*®-488 (both shown in red). For staining control, cells were also treated only with the respective secondary antibodies (right row, C+F). Nuclei were counterstained with DAPI. An additional co-staining of F-actin was performed with Alexa 568®-labeled phalloidin (here in green), shown in the lower panels (D-F). Fluorescence micrographs were taken with a confocal laser scanning microscope (Zeiss LSM 780) using a 63x/1.4 objective. The results suggest that treatment with ACT reduced the cell growth as well as the detected amount of S1PR1 in the cells.

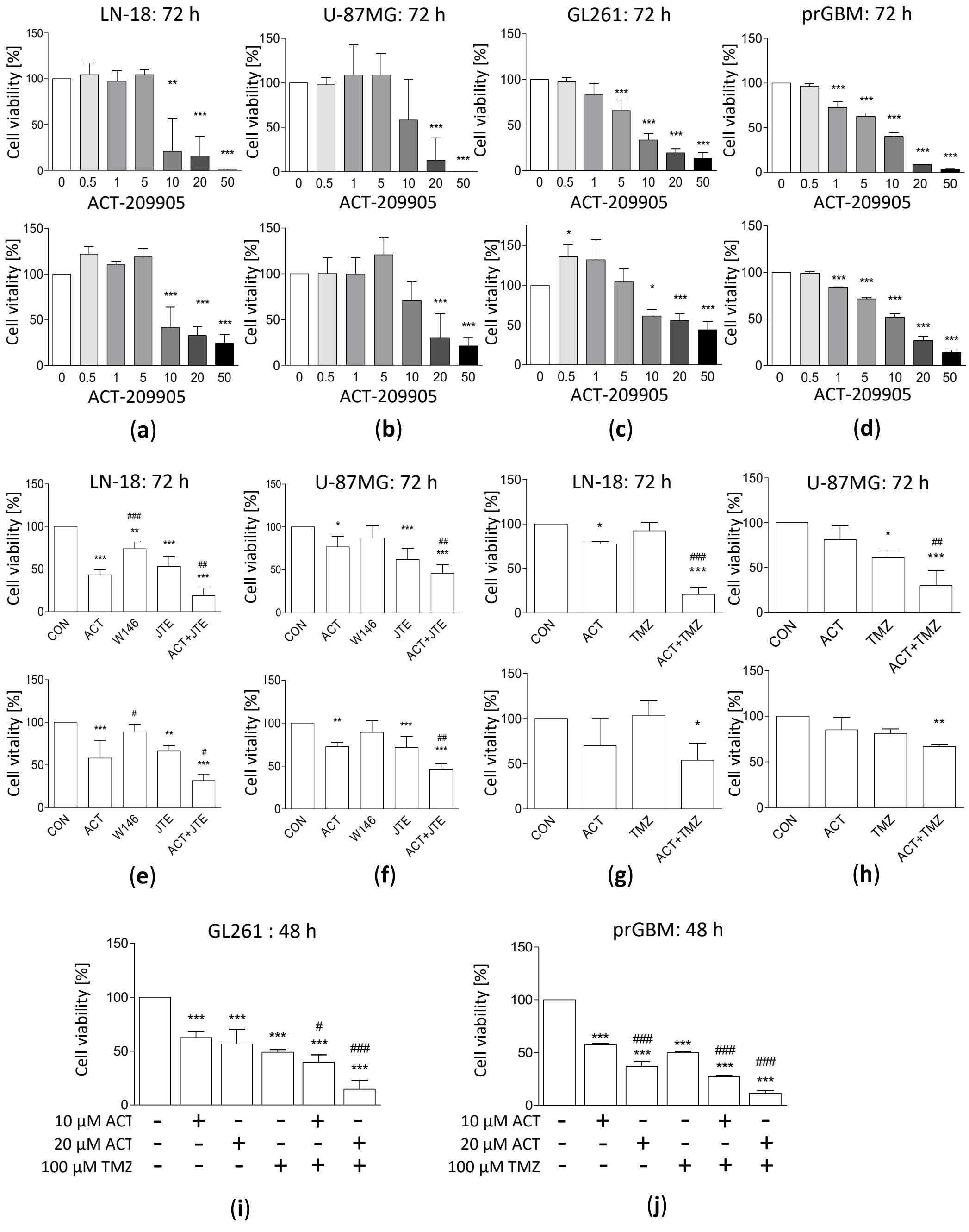

Supplemental Figure S2. Influence of S1PR1 modulator ACT-209905 alone or in combination with other compounds on cell viability of GBM cells. (a-d) Determination of cell viability with resazurin assay (upper panel) and cell vitality by crystal-violet staining (lower panel) for LN-18 (a), U-87MG (b), GL261 (c) and prGBM (d) cells after treatment with ACT-209905 (0.5, 1, 5, 10, 20 and 50 µM) for 72h. (e-f) Cell viability (resazurin assay, upper panel) and vitality (crystal-violet staining, lower panel) of LN-18 (e) and U-87MG (f) cells after application of ACT-209905 (10 µM), W146 (10 µM) and JTE-013 (10 µM) alone or in combination. (g-h) Cell viability (resazurin assay, upper panel) and vitality (crystal-violet staining, lower panel) after treatment of LN-18 (g) and U-87MG (h) cells with ACT-209905 (10 µM) alone or in combination with temozolomide (TMZ, 100 µM). (i-j) Cell viability (resazurin assay, upper panel) and vitality (crystal-violet staining, lower panel) after treatment of GL261 (i) and prGBM (j) cells with ACT-209905 (10 µM or 20 µM) alone or in combination with temozolomide (TMZ, 100 µM). Cell viability or vitality (d-j) was related to control treated cells (100%, only solvent), mean values and SD, n=3-5, one-way analysis of variance with Dunnett´s multiple comparison test or Bonferroni post-hoc test, *p<0.05, **p<0.01 and ***p<0.001 vs control (only solvent), ##p<0.01 and ###p<0.001 vs. 10 µM ACT-209905 alone.

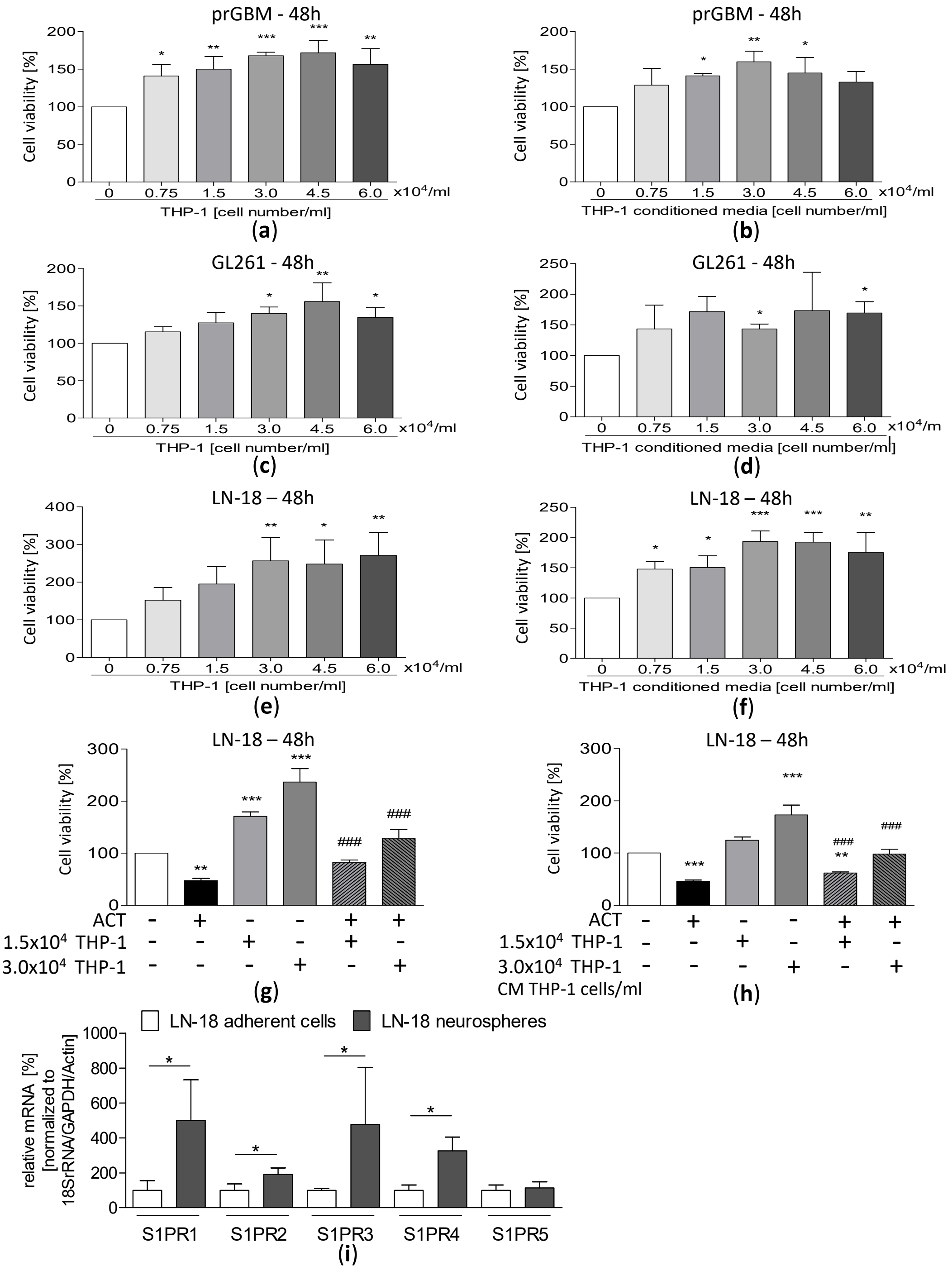

Supplemental Figure S3. Co-culture effect of THP-1 cells or conditioned media on viability of GBM cells after 48 h, and expression of S1P receptors on stem-like GBM neurospheres. GBM cells were co-cultured with THP-1 cells at the indicated cell densities or THP-1 conditioned medium for 48 h, and the viability of the cells was measured by the resazurin assay. (a+b) prGBM cells co-cultured with THP-1 cells (a) or THP-1 conditioned medium (b) for 48 h, (c+d) GL261 cells co-cultured with THP-1 cells (c) or THP-1 conditioned medium (d) for 48 h, and LN-18 cells co-cultured with THP-1 cells (e) or THP-1 conditioned medium (f) for 48 h; the viability of the cells was measured by the resazurin assay. (g+h) Co-cultivation of LN-18 cells with THP-1 cells (g) or THP-1 conditioned medium (h) in the presence of ACT-209905 (10 µM) after 48 h. Cell viability was determined using the resazurin assay and related to control cells (100%), mean values and SD, n=3-4, one-way analysis of variance with Dunnett´s multiple comparison test or Bonferroni post-hoc test, *$p<0.05$, **$p<0.01$ and ***$p<0.001$ vs control; #$p<0.05$, ##$p<0.01$ and ###$p<0.001$ vs 10 µM ACT-209905 alone. (I) mRNA expression of S1P receptors in LN-18 adherent cells in comparison to LN-18 neurospheres determined by quantitative RT-PCR. Mean values and SD, n=3, pairwise comparisons were performed using Mann–Whitney U test.

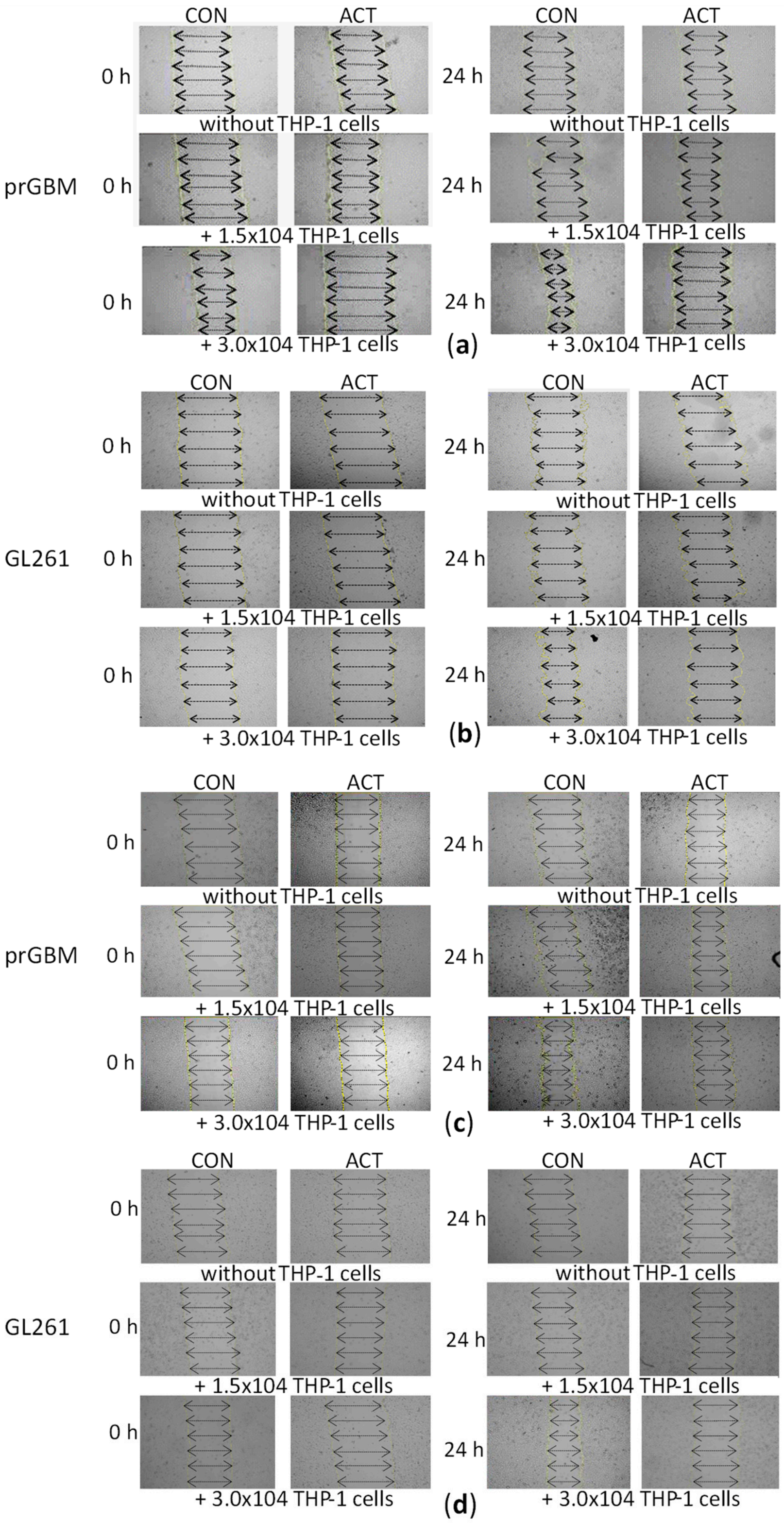

Supplemental Figure S4. Impact of THP-1 cells or THP-1 conditioned medium together with ACT-209905 application on migration of GBM cells. Co-culture of prGBM cells (a) or murine GL261 cells (b) with THP-1 cells alone or together with application of ACT-209905 (10 μM) and analyses of undirected migration using a wound closure assay by performing a scratch into the cell layer and measuring the wound width at the beginning of the experiment and after 24h of co-culture. (c+d) Co-culture of prGBM cells (c) or murine GL261 cells (d) with THP-1 conditioned media alone or together with application of ACT-209905 (10 μM) and analyses of undirected migration using a wound closure assay by performing a scratch into the cell layer and measuring the wound width at

the beginning of the experiment and after 24h of co-culture. Representative microscopy images of the scratch area. The statistical analysis and respective graphs are shown in Figure 4c-f.

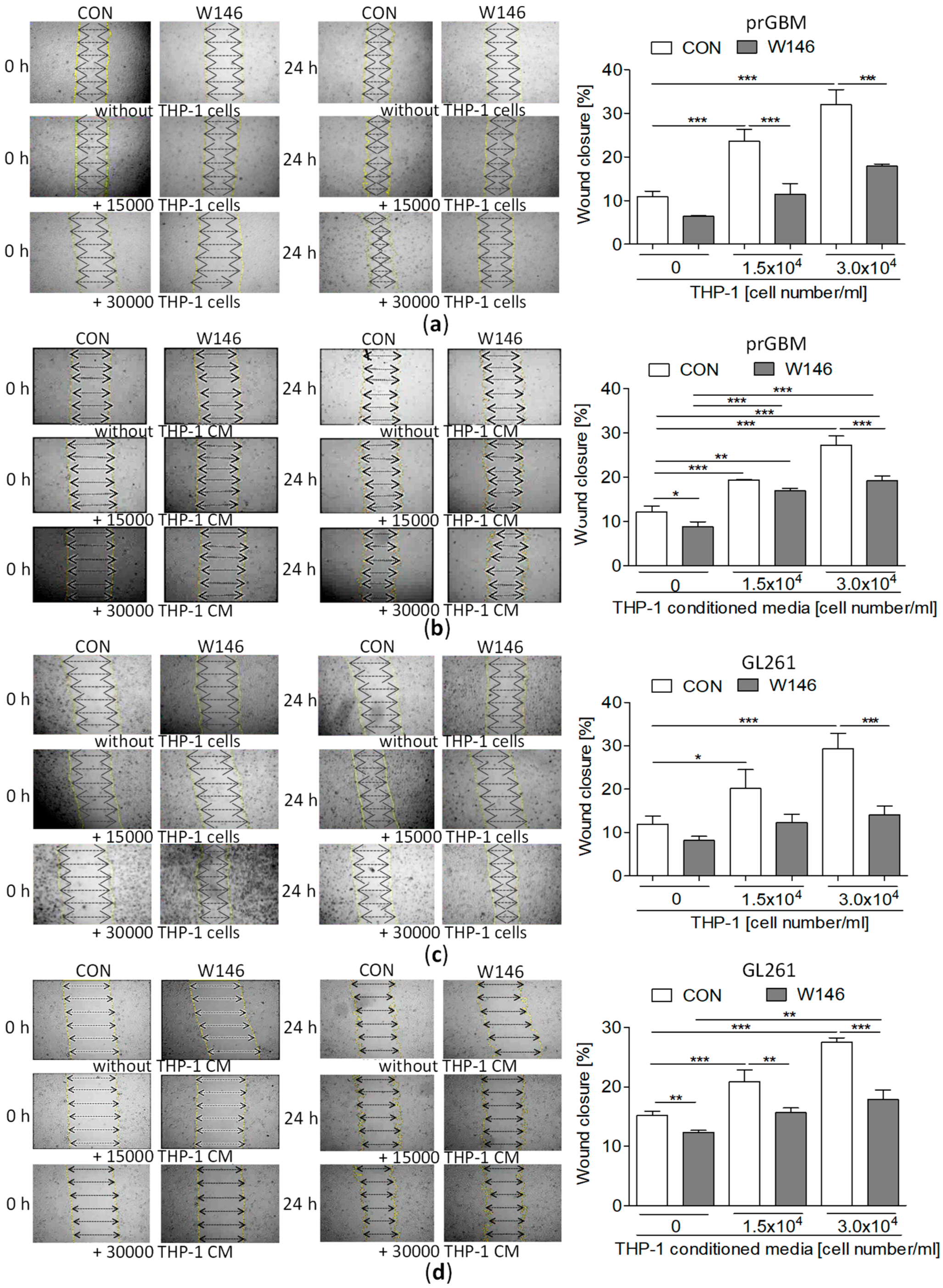

Supplemental Figure S5. Impact of THP-1 cells or THP-1 conditioned medium together with the S1PR1 antagonist W146 application on migration of GBM cells. (a+c) Co-culture of prGBM cells (a) or murine GL261 cells (c) with THP-1 cells alone or together with application of W146 (10 µM) and analyses of undirected migration using a wound closure assay by performing a scratch into the cell layer and measuring the wound width at the beginning of the experiment and after 24 h of co-culture. (b+d) Co-culture of prGBM cells (b) or murine GL261 cells (d) with THP-1 conditioned media alone or together with application of W146 (10 µM) and analyses of undirected migration using a wound closure assay by performing a scratch into the cell layer and measuring the wound width at the beginning of the experiment and after 24 h of co-culture. Representative microscopy images of the scratch area are shown. Mean values and SD, n=3, one-way analysis of variance with Bonferroni post-hoc test, *p<0.05, **p<0.01 and ***p<0.001 vs control.
