# Supplementary material for: The Putative S1PR1 Modulator ACT-209905 Impairs Growth and Migration of Glioblastoma Cells In Vitro
Source: Cancers (Basel). 2023 Aug 26;15(17):4273. doi: 10.3390/cancers15174273 (PMC10486705; doi:10.3390/cancers15174273)

Uncropped Blots from those shown in Figure 1a and 1b

Article

Modulation of Sphingosine-1-phosphate receptor 1 by ACT-209905 impairs growth and migration of Glioblastoma cells *in vitro*

Sandra Bien-Möller[1], Fan Chen[2], Yong Xiao[3], Sascha Marx[4], Celine Tolksdorf[5], Ulrike Meyer[6], Mladen V. Tzvetkov[7], Henry W. S. Schroeder[8], Bernhard H. Rauch[9]*

S1PR1 in different GBM cell lines and THP-1 cells

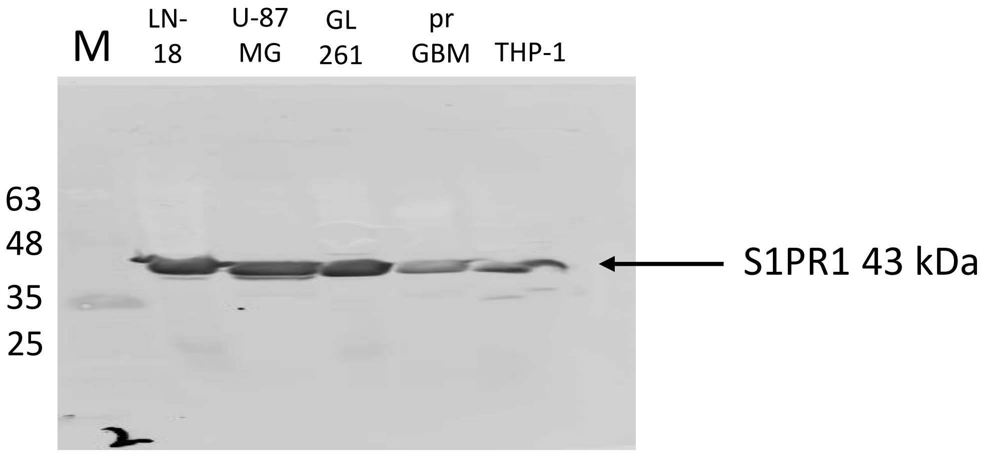

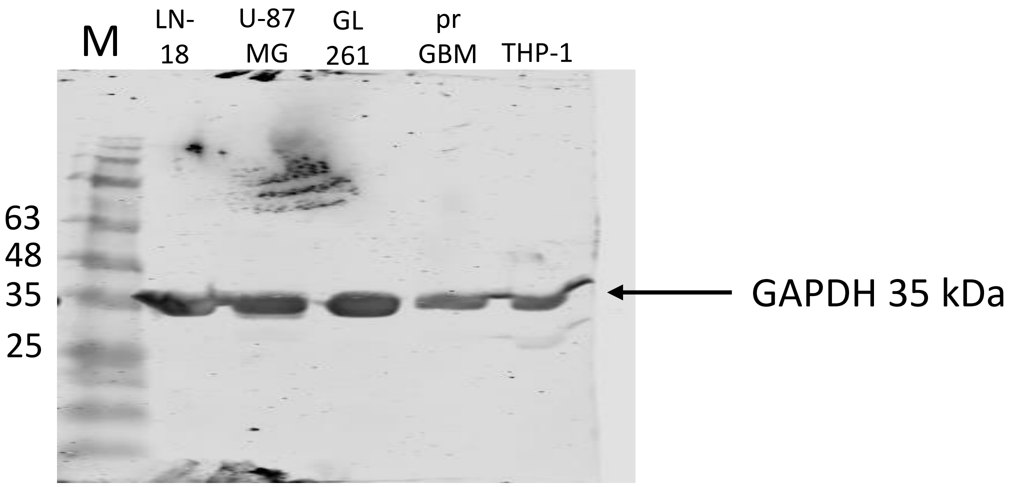

CD133, Nestin and GFAP in prGBM cells

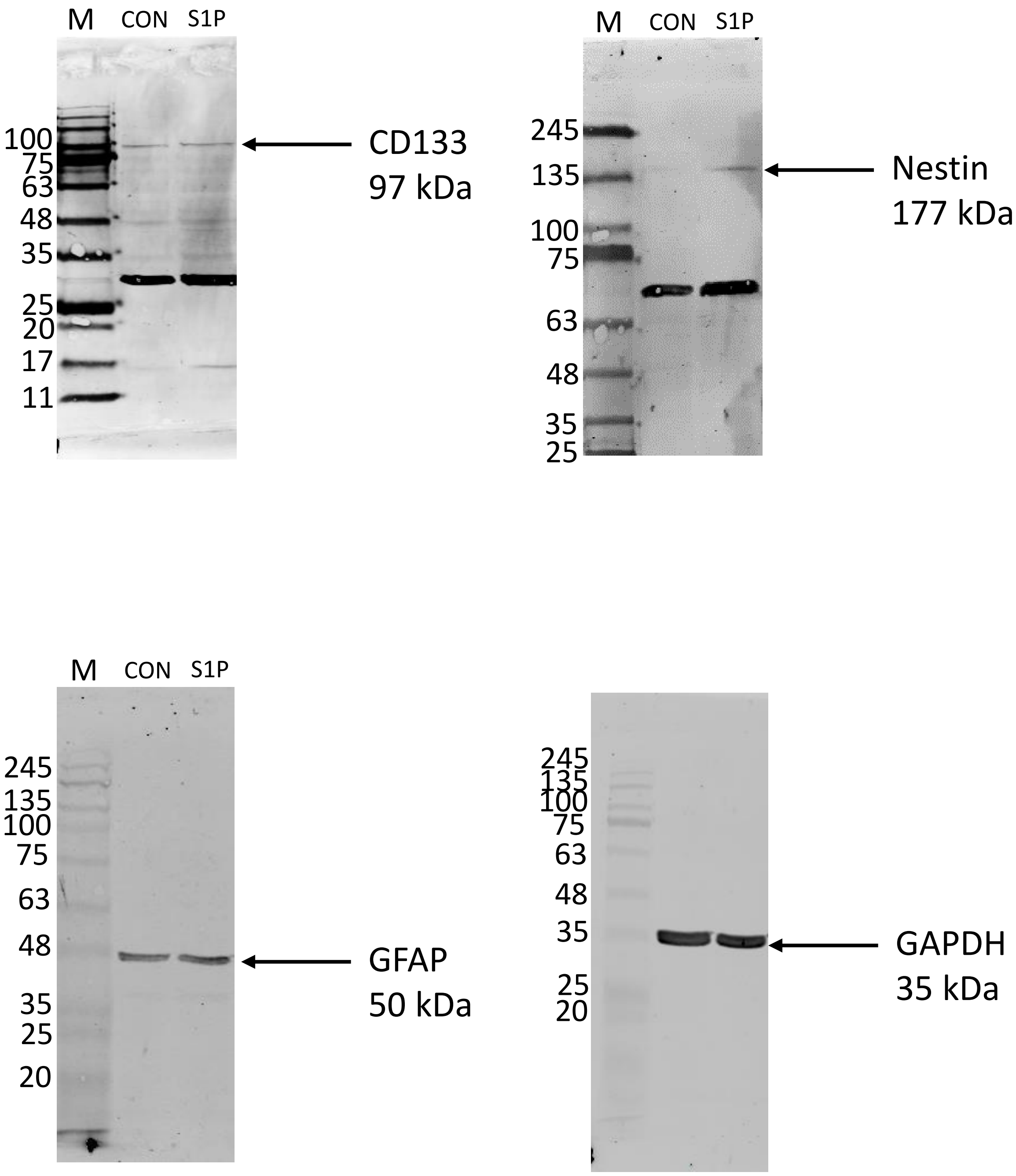

Uncropped Blots from those shown in Figure 6

Article

Modulation of Sphingosine-1-phosphate receptor 1 by ACT-209905 impairs growth and migration of Glioblastoma cells *in vitro*

Sandra Bien-Möller[1], Fan Chen[2], Yong Xiao[3], Sascha Marx[4], Celine Tolksdorf[5], Ulrike Meyer[6], Mladen V. Tzvetkov[7], Henry W. S. Schroeder[8], Bernhard H. Rauch[9]*

prGBM + THP-1 cells: PCNA

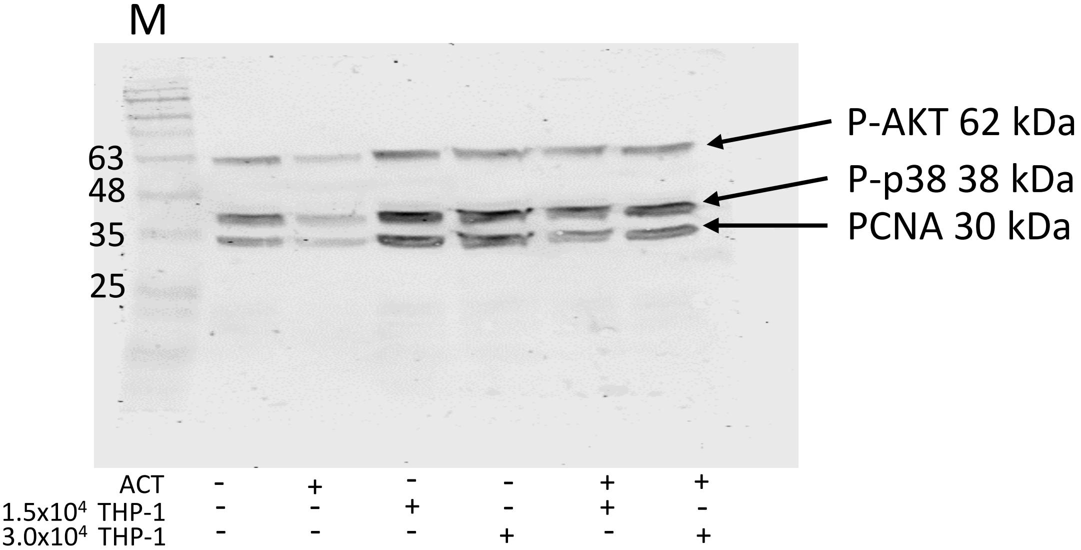

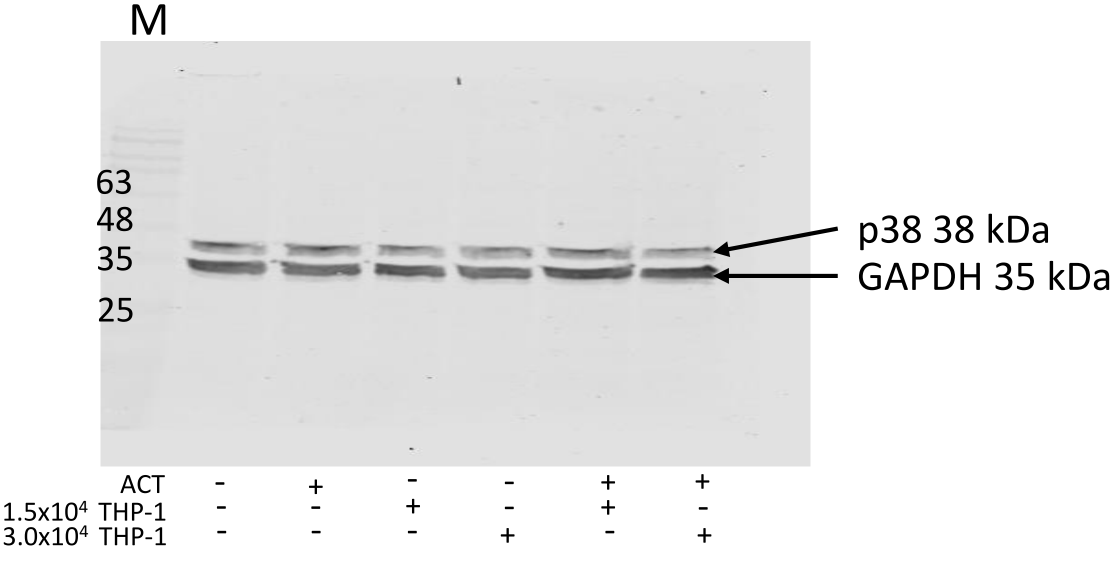

prGBM + THP-1 cells: p38

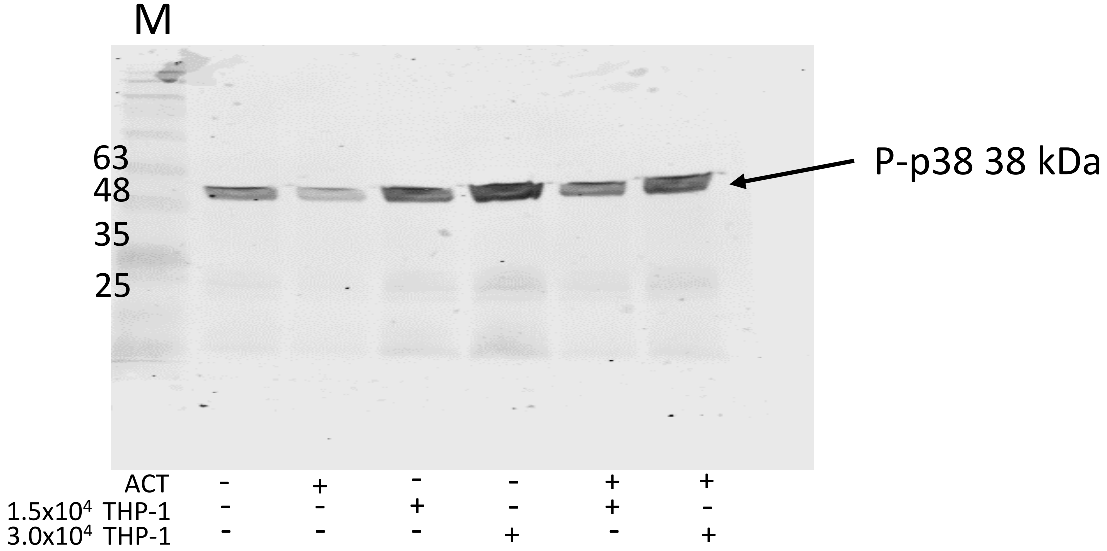

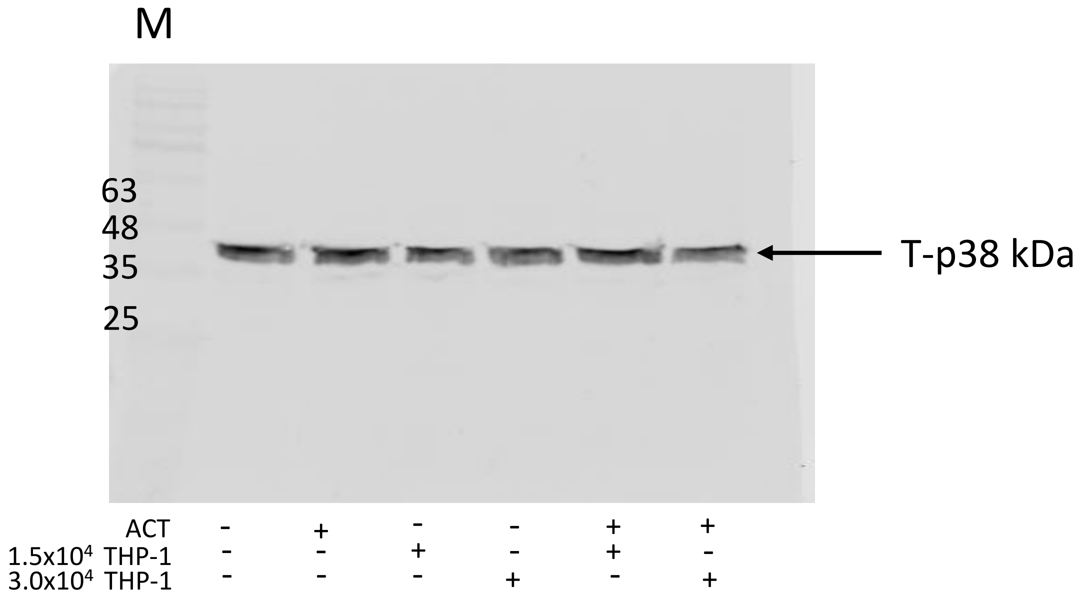

prGBM + THP-1 cells: AKT

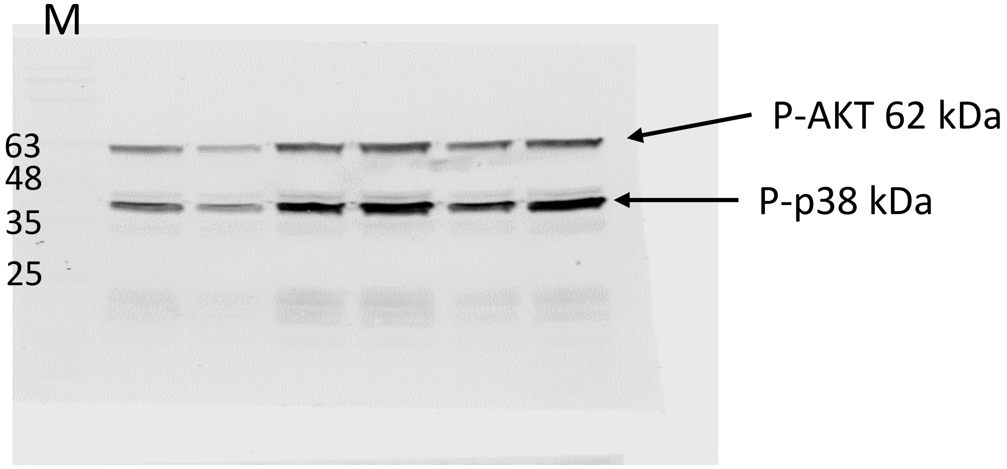

ACT	-	+	-	-	+	+
1.5x10⁴ THP-1	-	-	+	-	+	-
3.0x10⁴ THP-1	-	-	-	+	-	+

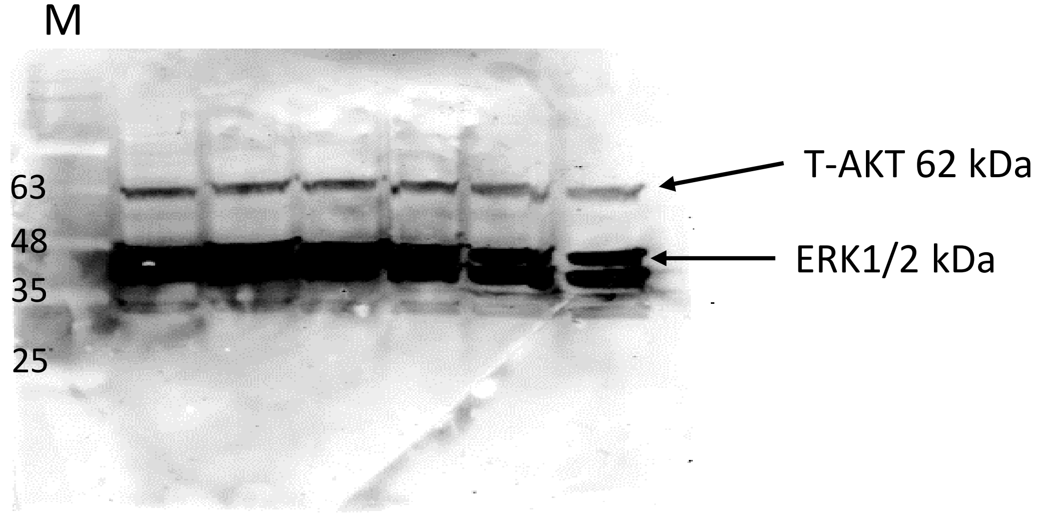

ACT	-	+	-	-	+	+
1.5x10⁴ THP-1	-	-	+	-	+	-
3.0x10⁴ THP-1	-	-	-	+	-	+

<u>prGBM + THP-1 cells: ERK1/2</u>

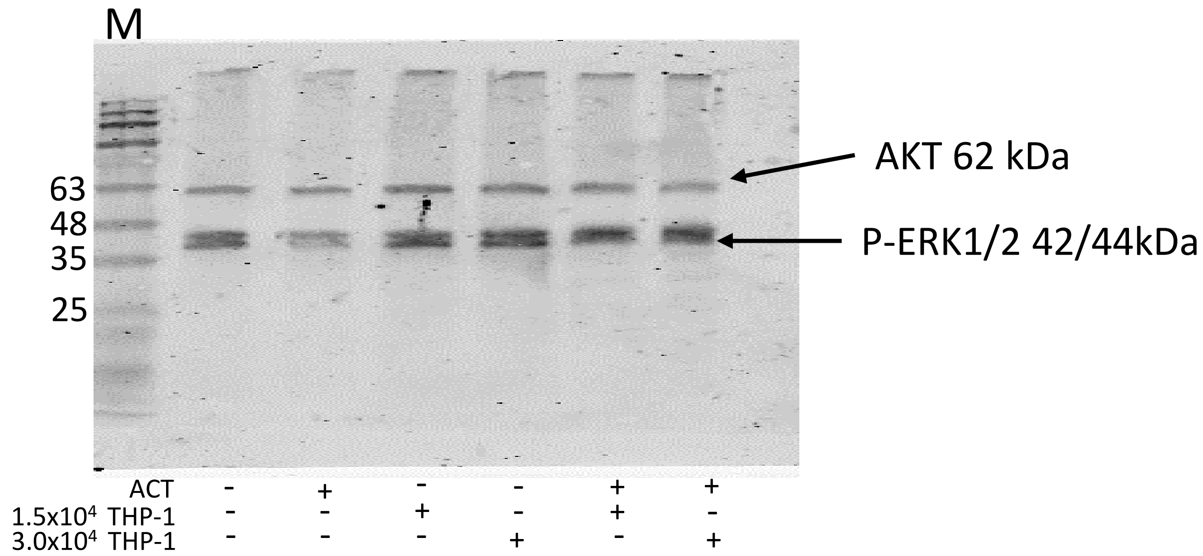

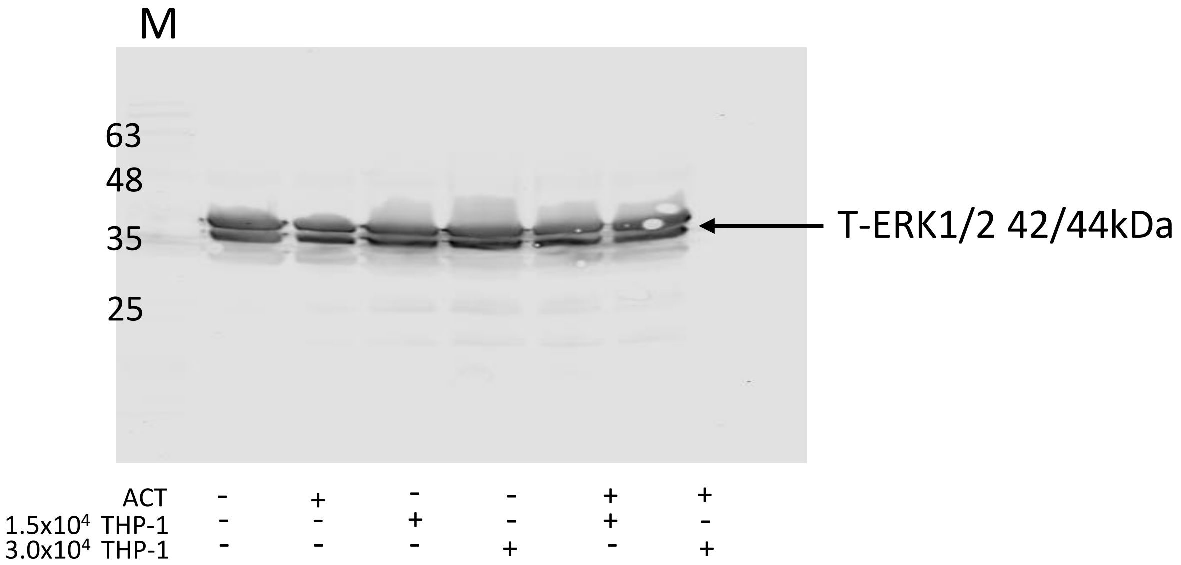

prGBM + THP-1 conditioned medium: PCNA

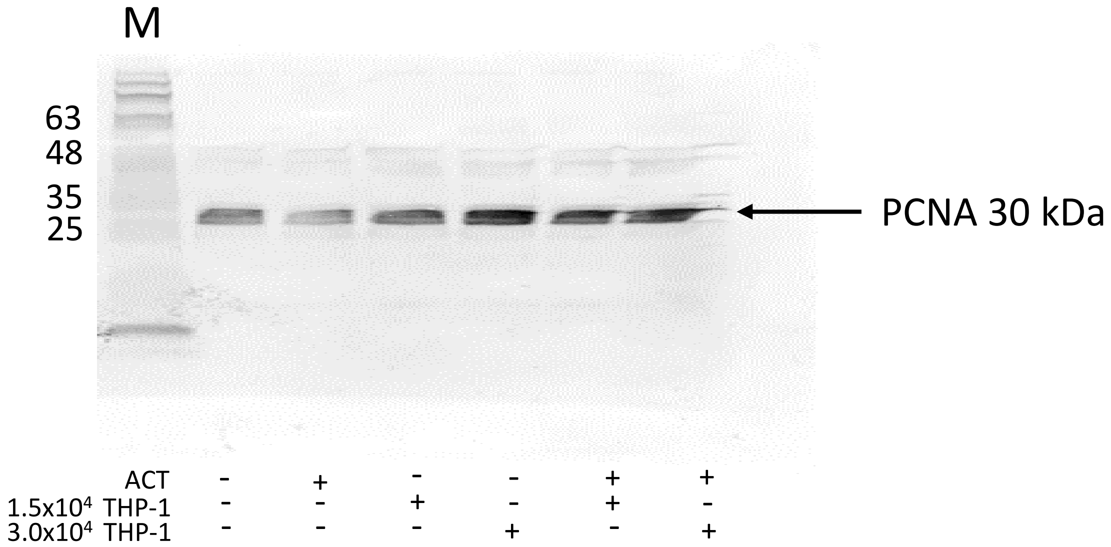

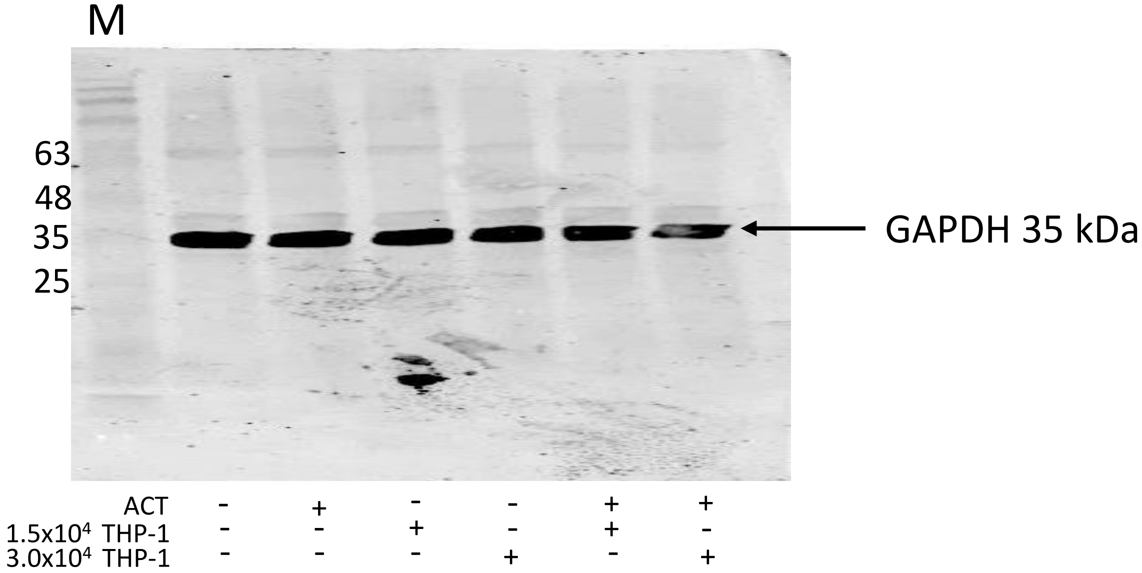

<u>prGBM + THP-1 conditioned medium: p38</u>

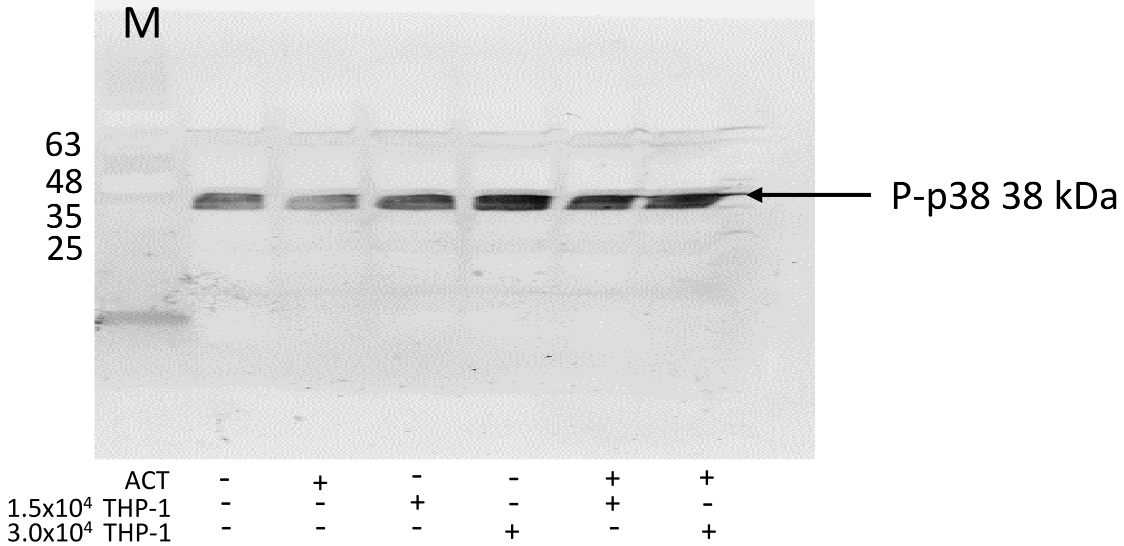

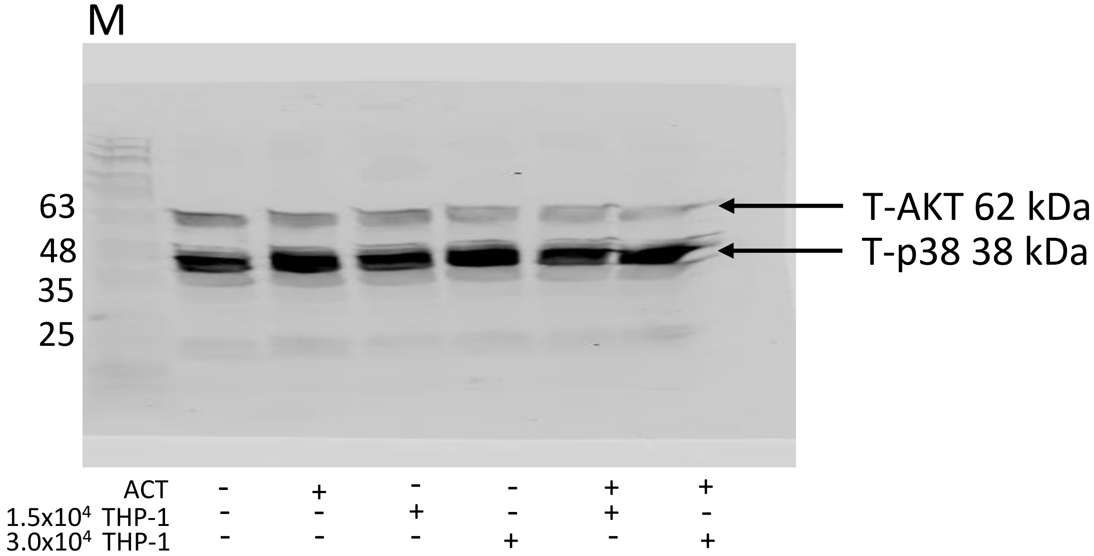

prGBM + THP-1 conditioned medium: AKT

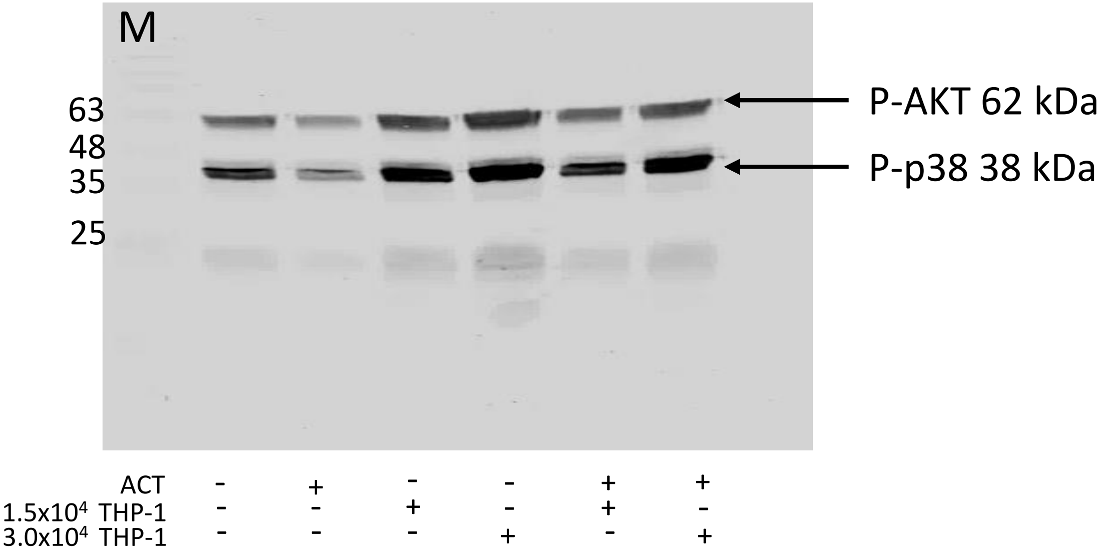

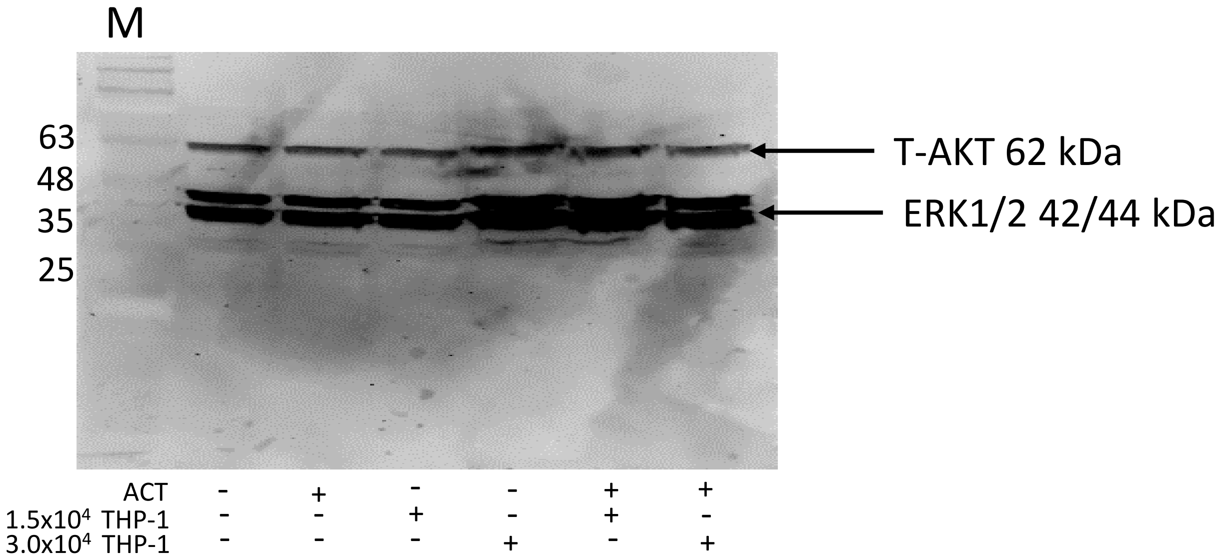

prGBM + THP-1 conditioned medium: ERK1/2

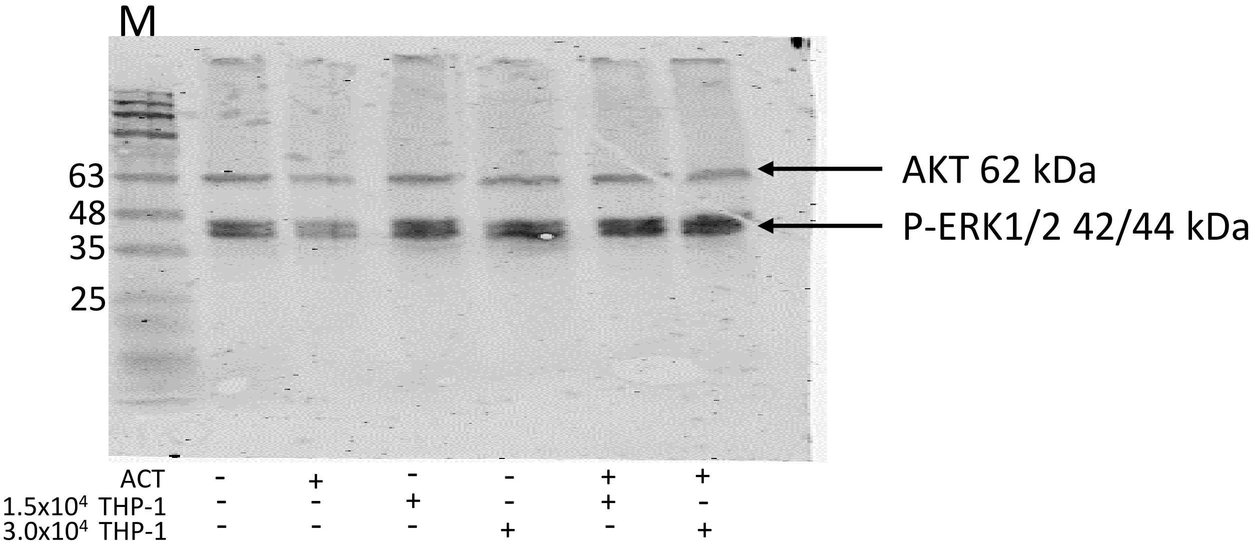

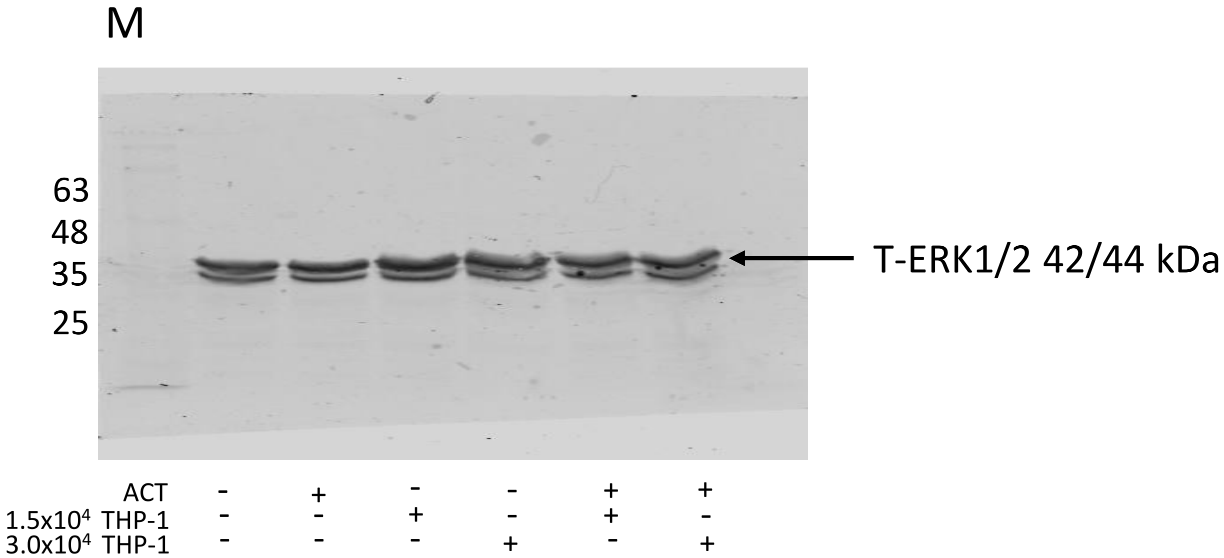

GL261 + THP-1 cells: PCNA

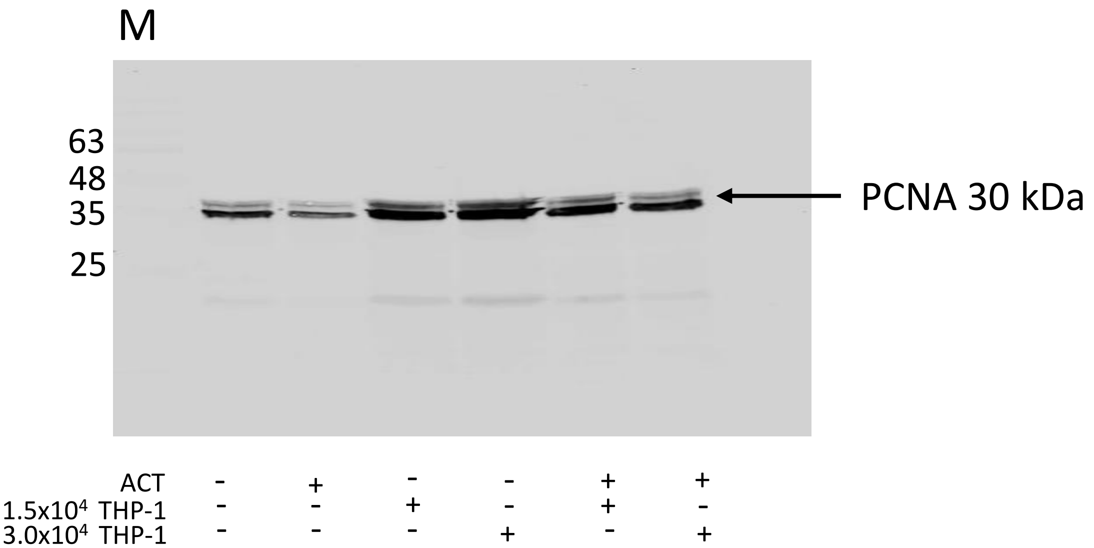

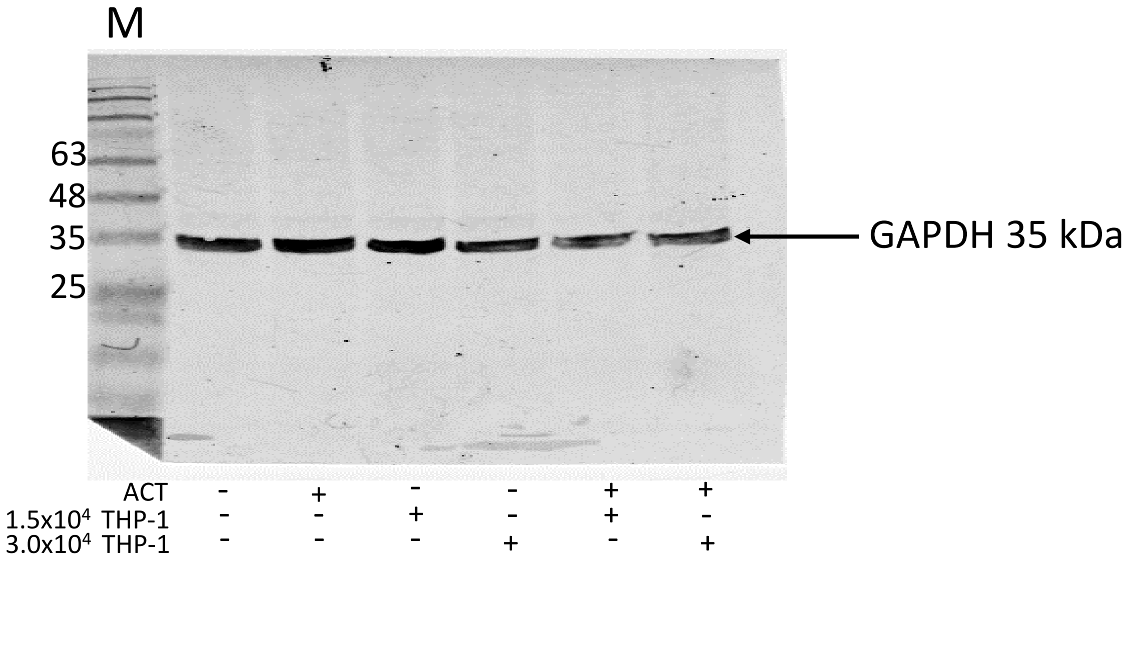

GL261 + THP-1 cells: p38

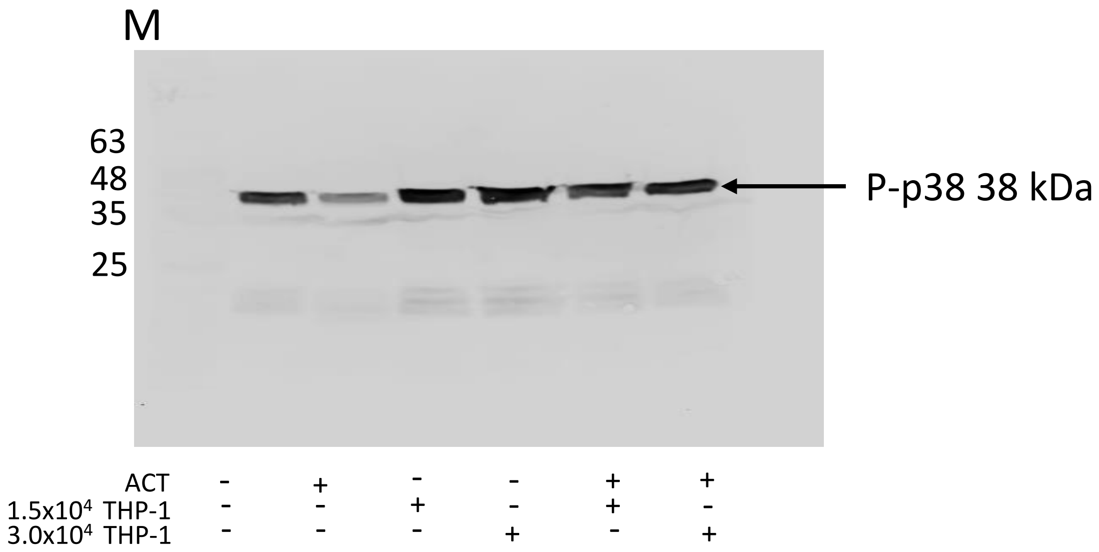

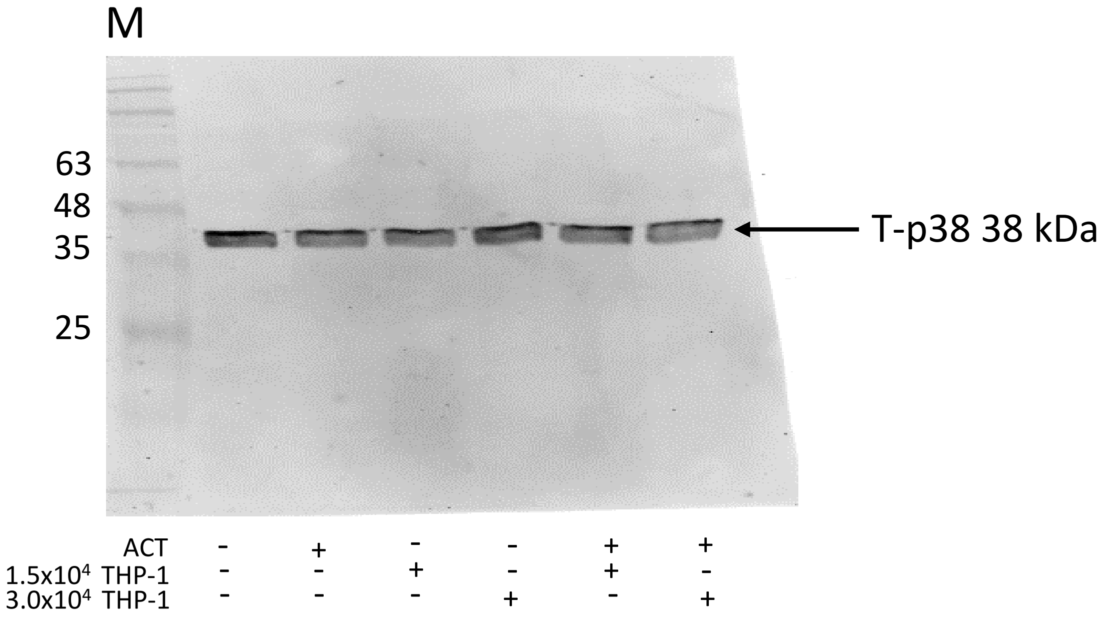

GL261 + THP-1 cells: AKT

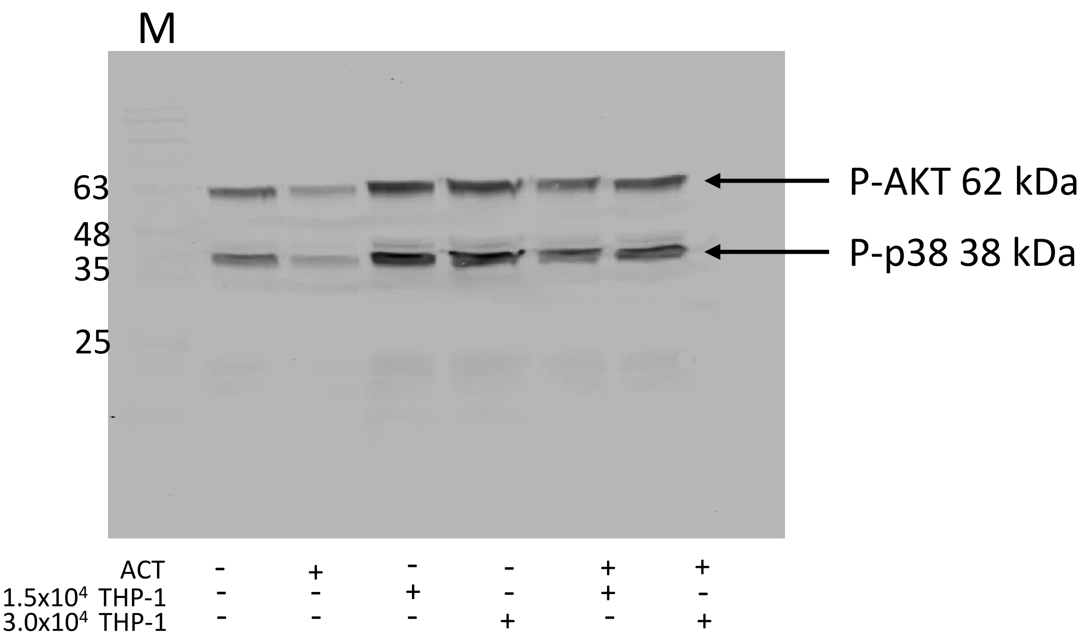

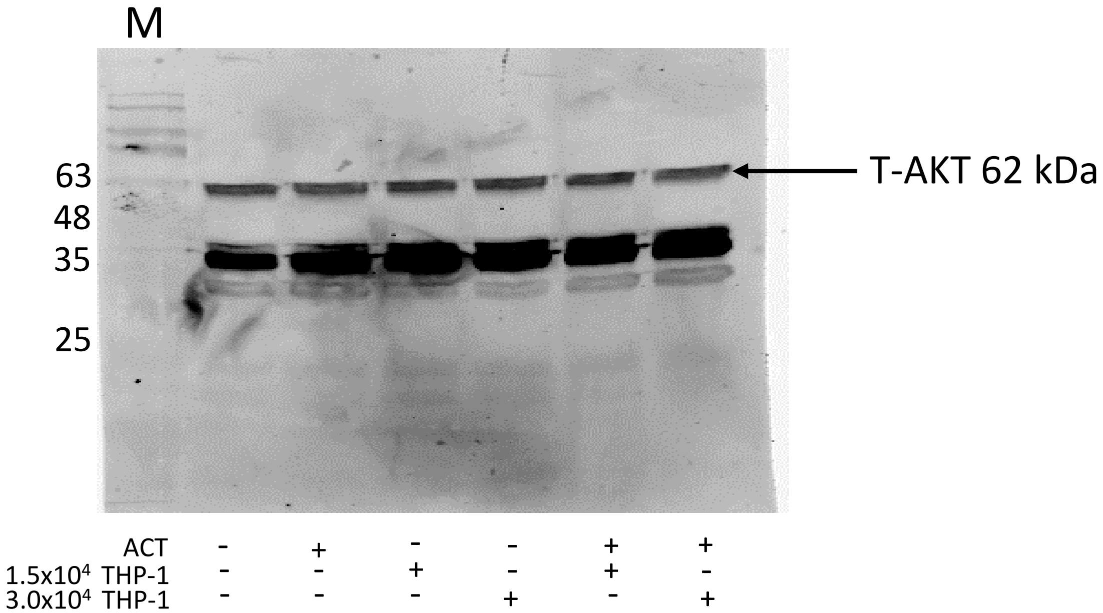

GL261 + THP-1 cells: ERK1/2

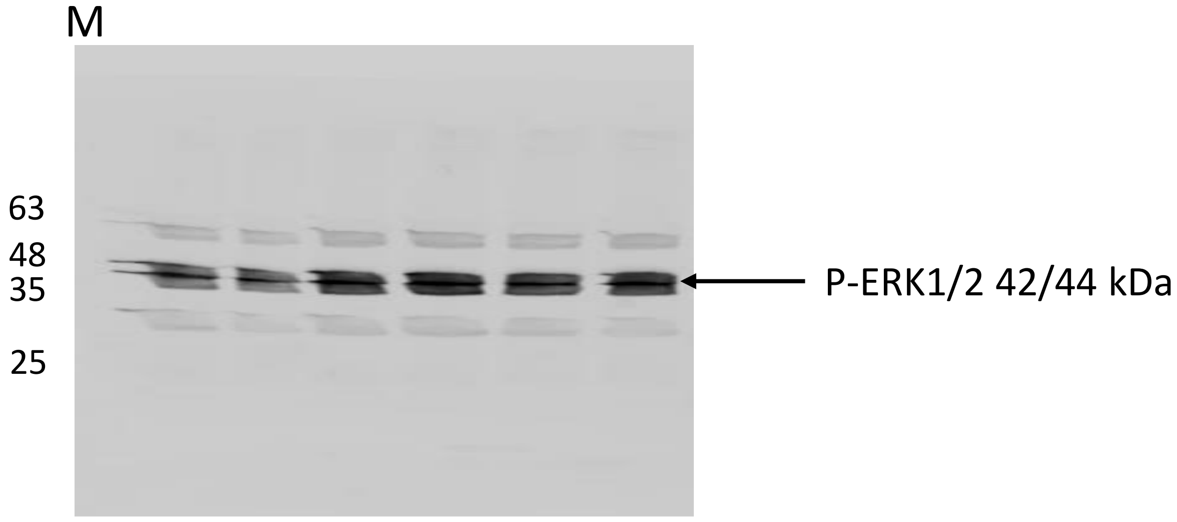

ACT	-	+	-	-	+	+
1.5x10^4 THP-1	-	-	+	-	+	-
3.0x10^4 THP-1	-	-	-	+	-	+

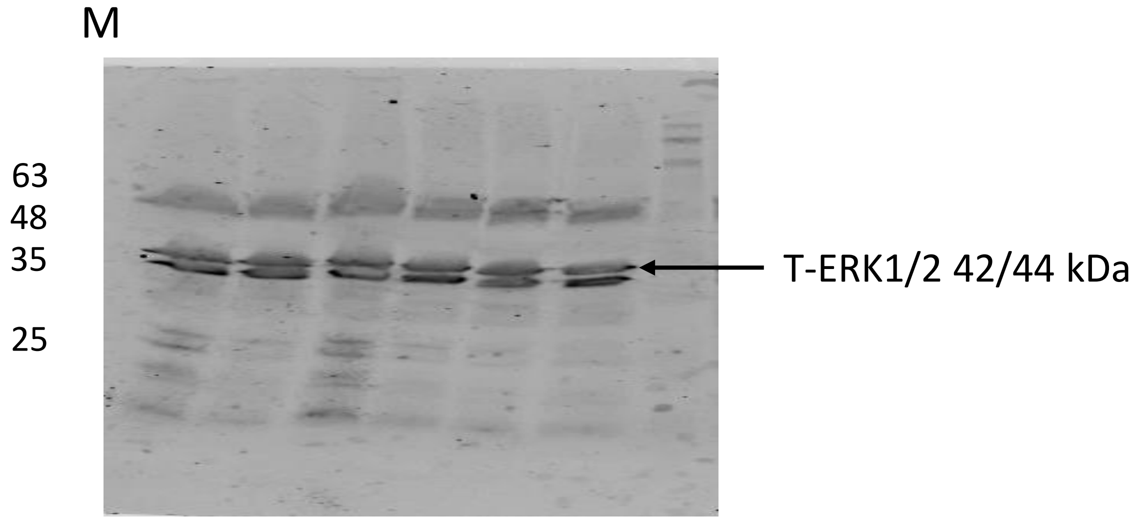

ACT	-	+	-	-	+	+
1.5x10^4 THP-1	-	-	+	-	+	-
3.0x10^4 THP-1	-	-	-	+	-	+

GL261 + THP-1 conditioned medium: PCNA

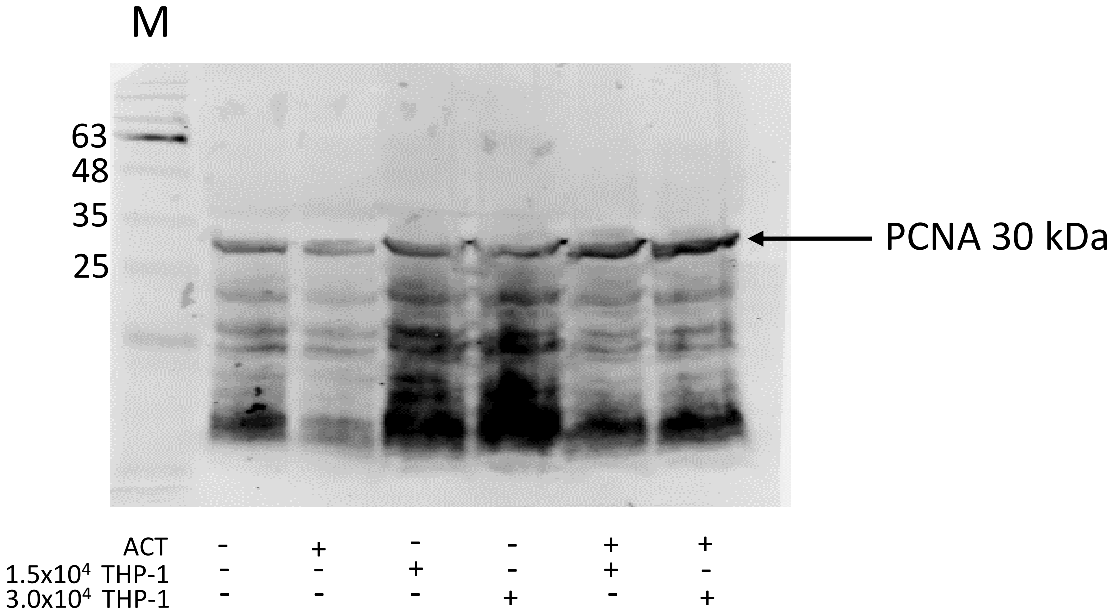

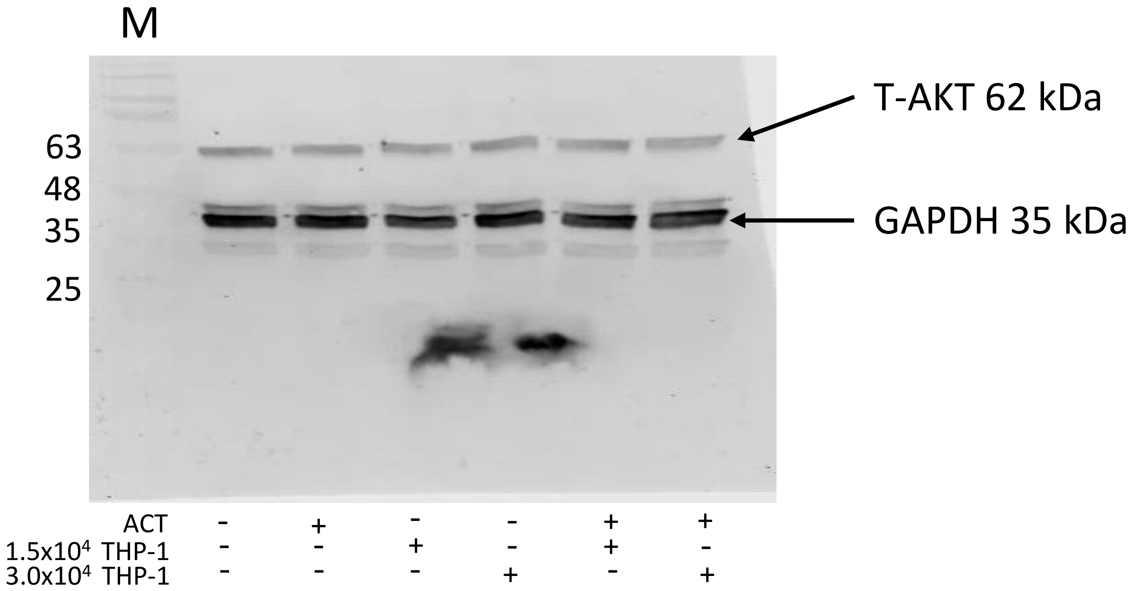

GL261 + THP-1 conditioned medium: p38

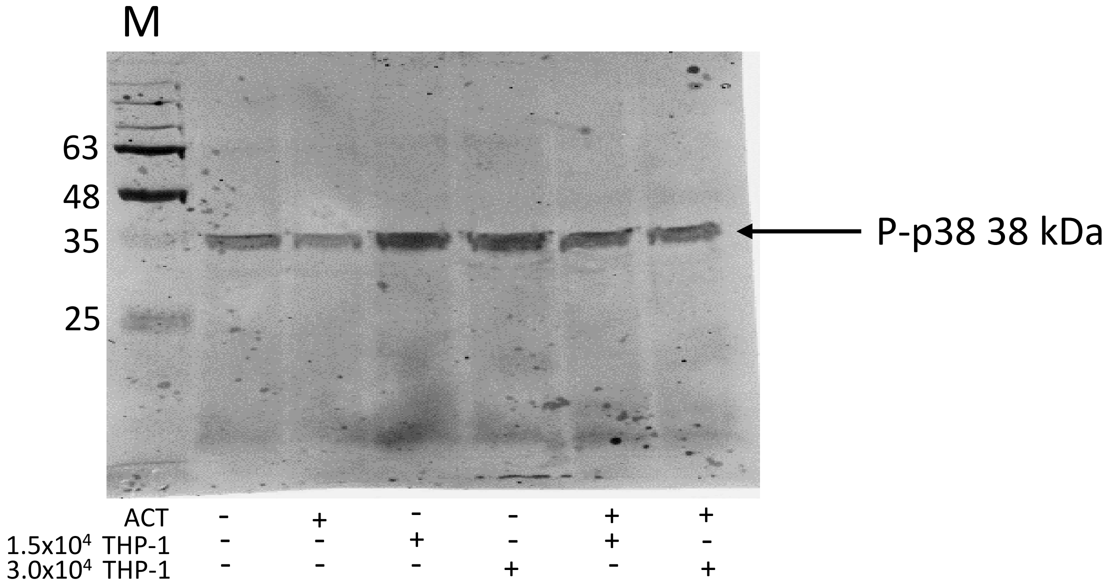

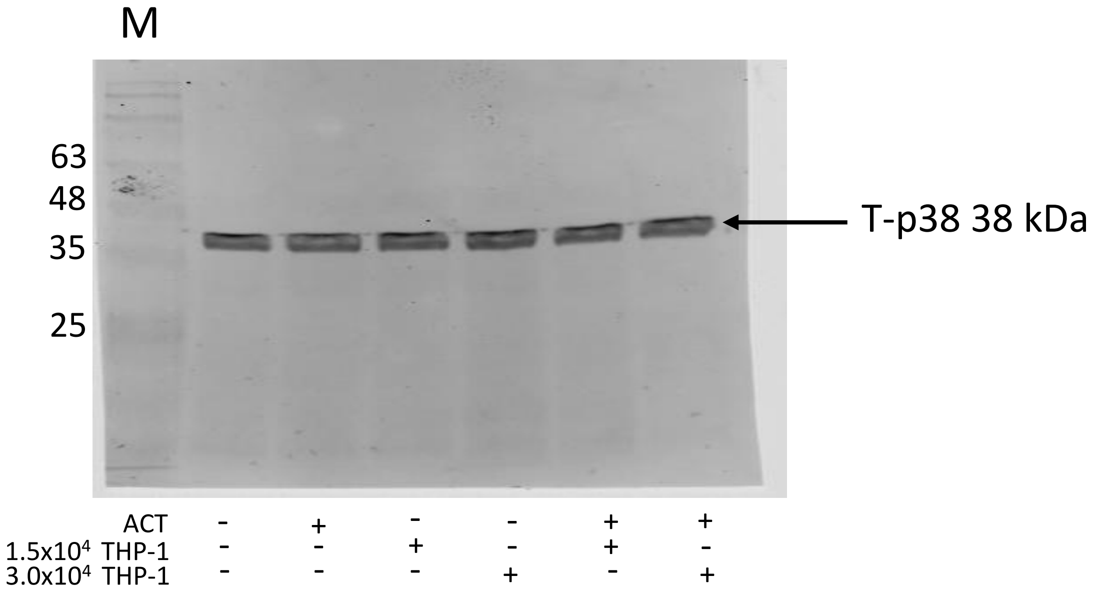

GL261 + THP-1 conditioned medium: AKT

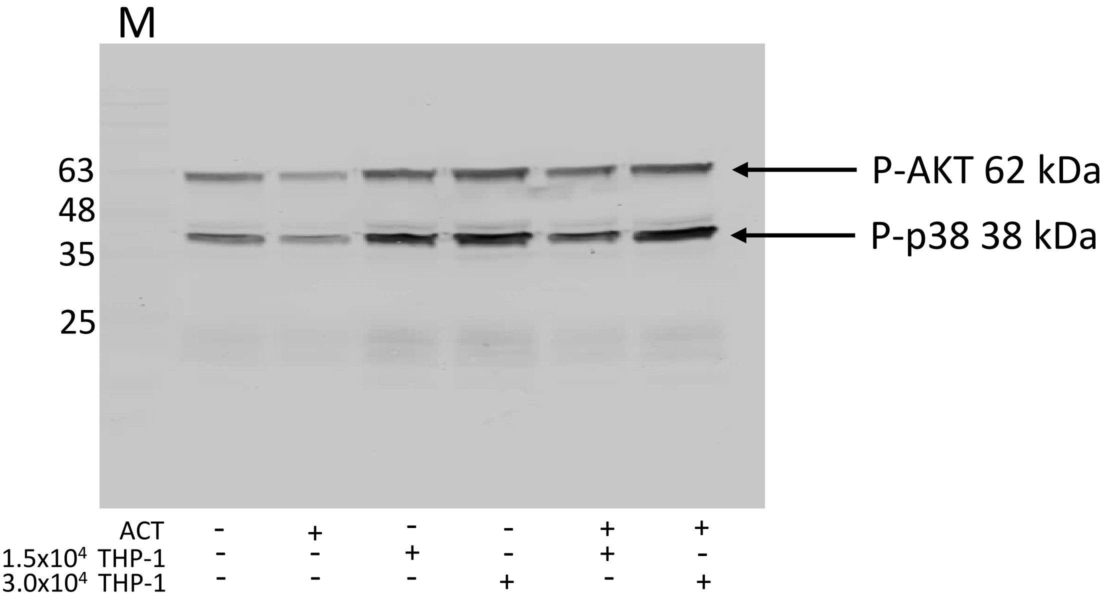

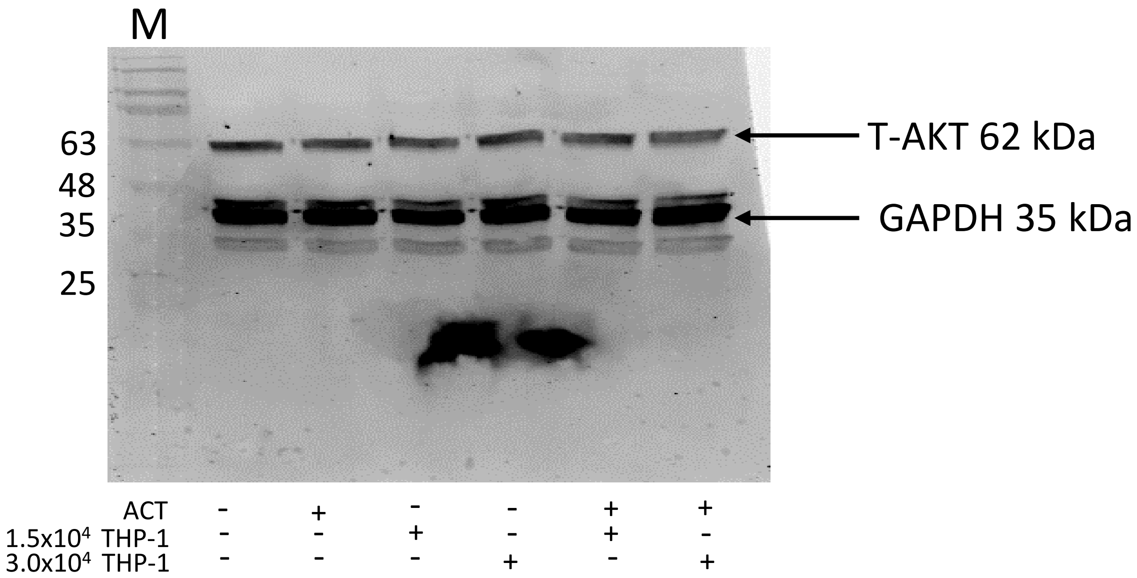

GL261 + THP-1 conditioned medium: ERK1/2

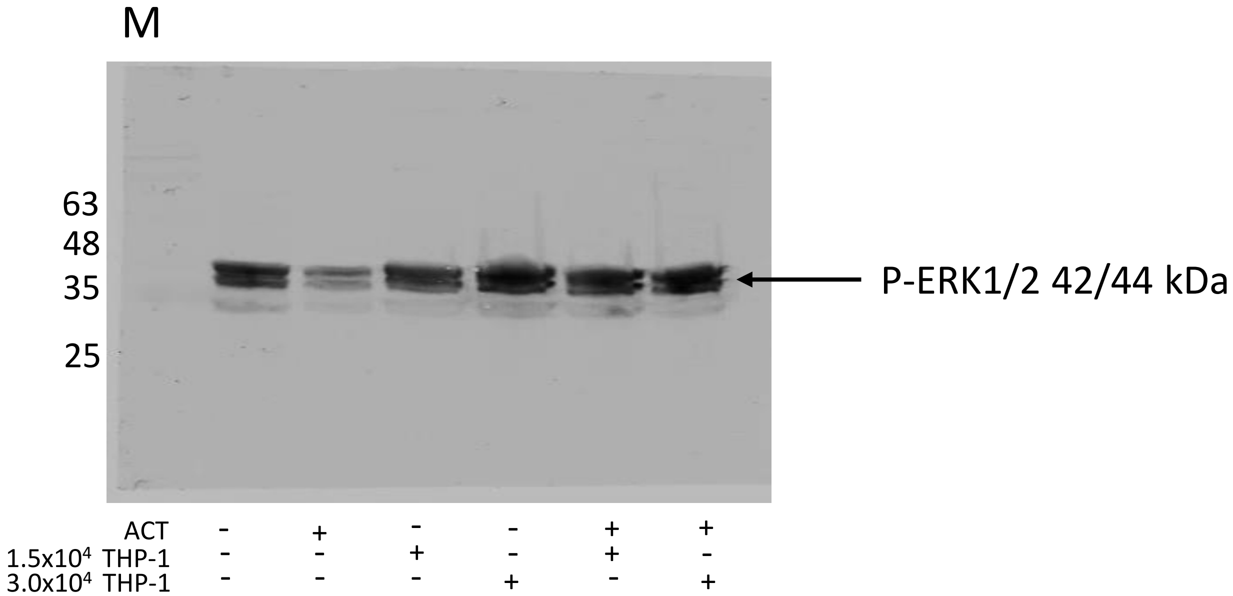

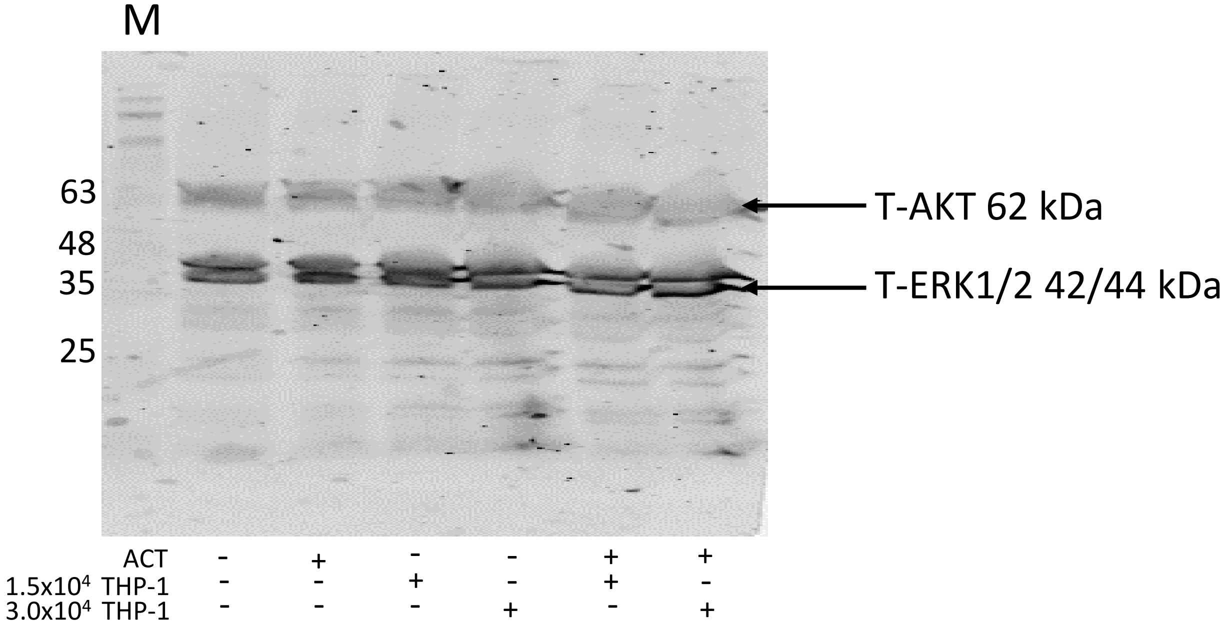

Supplement: Supplementary file 1 [file cancers-15-04273-s001.zip › Supplementery File S1.pdf]
